# Nuclear Expression Loss of SSBP2 Is Associated with Poor Prognostic Factors in Colorectal Adenocarcinoma

**DOI:** 10.3390/diagnostics10121097

**Published:** 2020-12-16

**Authors:** Yumin Chung, Hyunsung Kim, Seongsik Bang, Kiseok Jang, Seung Sam Paik, Su-Jin Shin

**Affiliations:** 1 Department of Pathology, Kangbuk Samsung Hospital, Sungkyunkwan University College of Medicine, Seoul 03181, Korea; umin0501@naver.com; 2 Department of Pathology, Hanyang University College of Medicine, Seoul 04763, Korea; hhnt5841@gmail.com (H.K.); grypony@naver.com (S.B.); medartisan@hanyang.ac.kr (K.J.); 3 Department of Pathology, Gangnam Severance Hospital, Yonsei University College of Medicine, Seoul 06273, Korea

**Keywords:** SSBP2, colon cancer, immunohistochemistry, prognosis

## Abstract

Single-stranded DNA binding protein 2 (SSBP2) is involved in DNA damage response and may induce growth arrest in cancer cells, having a potent tumor suppressor role. SSBP2 is ubiquitously expressed and the loss of its expression has been reported in various tumor types. However, the correlation between SSBP2 expression and colorectal cancer (CRC) prognosis remains unclear. SSBP2 nuclear expression was evaluated immunohistochemically in 48 normal colonic mucosae, 47 adenomas, 391 primary adenocarcinomas, and 131 metastatic carcinoma tissue samples. The clinicopathological factors, overall survival (OS), and recurrence-free survival were evaluated, and associations with the clinicopathological parameters were analyzed in 391 colorectal adenocarcinoma patients. A diffuse nuclear SSBP2 expression was detected in all normal colonic mucosa and adenoma samples. SSBP2 expression loss was observed in 131 (34.3%) primary adenocarcinoma and 100 (76.3%) metastatic carcinoma samples. SSBP2 expression was significantly associated with poor prognostic factors, such as vascular invasion (*p* = 0.005), high pT category (*p* = 0.045), and shorter OS (*p* = 0.038), using univariate survival analysis. Nuclear SSBP2 expression loss was significantly observed in colorectal carcinoma and metastatic carcinoma tissues, being associated with poor prognostic factors. SSBP2 acts as a tumor suppressor and may be used as a CRC prognostic biomarker.

## 1. Introduction

Colorectal cancer (CRC) is a leading cause of cancer-related morbidity and mortality worldwide, accounting for approximately about 700,000 deaths per year. Based on the GLOBOCAN series of the International Agency for Research on Cancer (IARC), CRC is the third most common cancer (10% of the total number) in men and the second most common (9.2% of the total number) in women [1]. 

CRC is a heterogeneous disease that exhibits variable underlying molecular changes, with genetic instability. Two major mechanisms of genetic instability include chromosomal instability (CIN, the most common type) and microsatellite instability (MSI) [2]. CIN includes changes in the chromosome number and structure, such as deletions, gains, translocations, and other chromosomal rearrangements [3]. MSI occurs due to a defective DNA mismatch repair. For example, Lynch syndrome or hereditary nonpolyposis colorectal cancer syndrome is caused by inherited mutations in one of the mismatch repair (MMR) genes (predominantly *MLH1* and *MSH2*) [4]. Markowits et al. described the three most important molecular pathways for CRC development [5]. The first pathway involves genomic instability due to CIN, MSI, and aberrant DNA methylation. The second pathway involves mutational inactivation of tumor-suppressor genes, such as *APC*, *TP53*, and *TGF-β*. The third pathway involves the activation of oncogenes, such as *RAS*, *BRAF*, and *phosphatidylinositol 3-kinase*. Recently, promoter methylation has been studied in several human malignancies. Methylation leads to transcriptional silencing and plays a crucial role in the loss of expression of tumor suppressor or DNA repair genes [6,7]. Somatic mutations of the *adenomatous polyposis coli* (*APC*) tumor suppressor gene are common in sporadic CRC [8]. However, in up to 20% of CRC tumors, *APC* mutation is not present and gene inactivation via transcriptional silencing due to promoter hypermethylation led to the loss of *APC* function. Esteller et al. found that the *APC* promoter is hypermethylated in 18% of primary sporadic CRCs, and that methylation affected wild-type *APC* in 95% of cases [9].

Currently used tests in clinics related to prognosis and treatment in CRCs include MSI testing, mutations in *RAS*, and EGFR immunohistochemical test for anti-EGFR therapy. For example, MMR deficiency can be determined by performing immunohistochemical staining for the four major proteins MLH1, MSH2, MSH6, and PMS2 that make up the MMR system. In general, deficient mismatch repair (dMMR) is defined as when the nuclear expression of one or more MMR proteins (MLH1, PMS2, MSH2, or MSH6) is lost [10]. Approximately 20% of stage II and stage III CRCs exhibit a dMMR/MSI phenotype and are associated with a better prognosis than pMMR (proficient mismatch repair)/MSS (microsatellite stable) tumors [11]. Several studies have provided evidence that patients with dMMR tumors do not benefit from adjuvant chemotherapy [12,13,14]. Therefore, the MMR protein test plays an important role in decision making for treatment regimen.

Many studies have been conducted to find biomarkers related to the prognosis of CRCs. A recently published review article identified biomarkers associated with treatment response (PTEN, AREG, EREG, PI3K), metastasis and progression risk (ALDH1, *BRAF* V600E, CDX2, leptin, *c-MET*), and survival rate (ARID3A, FOXP3, HIF-1α, Ran) [15]. Although many biomarkers have been studied, most of them, except for *RAS* mutations and MSI status, are currently not clinically useful. Therefore, further research for the new clinically useful biomarker is needed.

The human *single stranded DNA binding protein 2* (*SSBP2*) gene was first identified in primary leukemic blasts and was found to be translocated and deleted in myelodysplasia and acute myelogenous leukemia (AML). SSBP2 expression has been detected in hematopoietic and non-hematopoietic tissues [16,17]. The loss of SSBP2 expression is associated with various types of malignancies, such as esophageal squamous cell carcinoma, prostate cancer, gallbladder cancer, and acute myeloid leukemia [18,19,20,21]. SSBP2 was shown to play a tumor-suppressive role in esophageal squamous cell carcinoma via inhibition of the Wnt signaling pathway [18]. In addition, Maldonado et al. found that a decreased SSBP2 expression was associated with an increased risk of recurrence in late stage prostate cancer [22]. Liang et al. described that the candidate myeloid leukemia suppressor gene encoding sequence-*SSBP2* from chromosome 5q13.3 was frequently deleted in AML. They identified the frequent loss of SSBP2 protein expression in human AML cell lines using highly specific antibodies. Surprisingly, the inducible expression of SSBP2 was accompanied by a downregulation of C-MYC expression. In addition, they briefly mentioned the possibility that SSBPs may directly repress C-MYC expression in CRC [13]. However, He et al. analyzed the association between C-MYC and CRC prognosis in a meta-analysis. They concluded that C-MYC was not associated with CRC prognosis [23]. Meanwhile, several studies on promoter methylation of *SSBP2* in tumors have shown that *SSBP2* is one of the genes that are downregulated by methylation [18,20,21,24,25].

The relationship between SSBP2 and CRC has been reported in a few studies. First, Andersen et al. described that SSBP2, a transcription factor upregulated by Wnt inactivation, was downregulated in CRC by performing quantitative real-time RT–PCR (qRT–PCR). However, validation using immunohistochemistry was not performed [26]. Second, Shannon et al. found that SSBP2 may be a potential biomarker, identifying it and optimizing it for immunohistochemistry (IHC) in CRC with peritoneal metastasis. Validation was performed on patients with colorectal peritoneal metastasis (CPM) that underwent CRS (cytoreductive surgery)-HIPEC (hyperthermic intraperitoneal chemotherapy) (*n* = 62), using IHC. They described that patients exhibiting a lower expression of SSBP2 potentially had a poorer overall survival (OS) and a shorter disease-free survival (DFS), compared to those with a higher expression, although they were not statistically significant [27]. Third, Perilli et al. found that an increased level of miR-182-5p (miR-182), one of the most upregulated oncogenic microRNAs (miRNAs) in CRC, was associated with a significant decrease in SSBP2 mRNA levels in the tumor tissues, compared to matched normal mucosa [28].

In this study, we examined the expression of SSBP2, its prognostic significance, and its association with the clinicopathological features in CRC patients.

## 2. Materials and Methods

### 2.1. Patients

We retrospectively collected data from patients with colorectal adenocarcinoma who underwent curative surgery at the Hanyang University Hospital, Seoul, between January 2005 and December 2010. A total of 391 patients were included after excluding patients who received neoadjuvant chemotherapy and/or radiation therapy, the ones who died within 30 days of surgery, or had insufficient tissue material for analysis. Primary colorectal adenocarcinoma tissues (*n* = 391), matched normal colonic mucosa tissues (*n* = 48; randomly selected from 391 cases), and matched metastatic carcinoma tissues (*n* = 131; 92 lymph node metastasis and 39 distant organ metastasis) were obtained from the colorectal adenocarcinoma patients. In addition, 40 patients diagnosed with adenoma with low-grade dysplasia who underwent biopsy or polypectomy at the Hanyang University Hospital, Seoul, between January 2013 and December 2014, were randomly selected. Four patients diagnosed with adenoma exhibited multiple adenomatous polyps, and 47 adenoma tissue samples were obtained from them. 

A total of 617 formalin-fixed, paraffin-embedded tissue samples were collected and 2.0-mm-core tissue microarray (TMA) blocks were constructed with one representative core for each case. The percentage of tumors in each cancer tissue core was greater than 70%. Clinical data, including age, sex, tumor location, tumor size, histologic grade, lymphovascular invasion, perineural invasion, tumor deposit, tumor budding, and TNM (tumor (T), nodes (N), and metastases (M)) staging were obtained from the medical records. Staging was determined according to the American Joint Committee on Cancer (8th edition) [29]. This study was approved by the Hanyang University Hospital (No. 2016-12-030-001), and the requirement for informed consent was waived.

### 2.2. Immunohistochemical Stainings and Interpretation

SSBP2 expression was evaluated using immunohistochemical staining of 4-μm-thick sections from TMA blocks. Rabbit monoclonal anti-SSBP2 antibody (1:100, ab177944, Abcam, Cambridge, UK) was used. The sections were first deparaffinized in xylene and then rehydrated through a graded ethanol series. For antigen retrieval, we heated the samples to 100 °C for 30 min in sodium citrate buffer (pH 6.0). Endogenous peroxidase activity was blocked using a peroxidase blocking solution (S2023, DakoCytomation, Carpinteria, CA, USA). TMA slides were incubated with primary antibodies at 4 °C overnight and then incubated with the labeled polymer (DAKO REAL EnVision/HRP, K5007, DakoCytomation, Glostrup, Denmark) for 30 min at room temperature. Then, 3,3-diaminobenzidine was used as a chromogen for visualization, and Mayer’s hematoxylin counterstain was applied.

**Figure 1:**
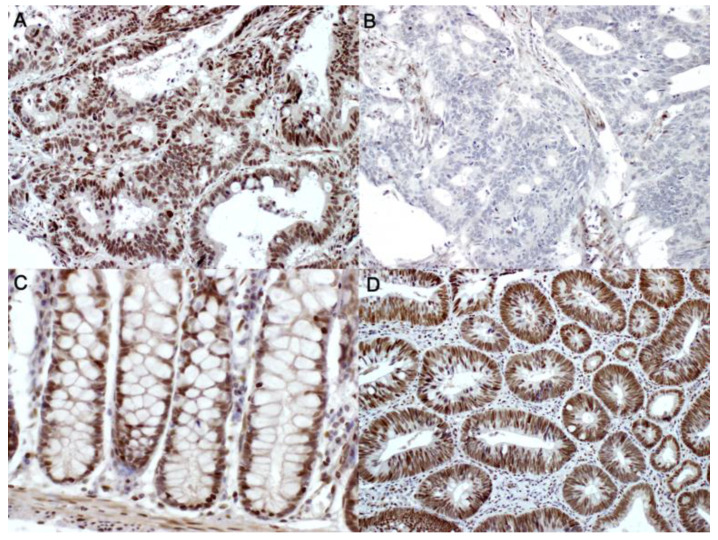
Immunohistochemical staining of single-stranded DNA-binding protein 2 (SSBP2). Representative figures of SSBP2-positive (**A**, ×200) and SSBP2-negative (**B**, ×200) colorectal adenocarcinoma, SSBP2-positive normal colonic tissues (**C**, ×200), and SSBP2-positive tubular adenoma (**D**, ×200).

The expression of SSBP2 was evaluated according to the extent of tumor cell nuclear staining, using a light microscope, by two pathologists (YM and SS) who were blinded to the clinical data. The patients were subsequently subdivided into negative (proportion of positive tumor cells ≤ 10% of the total tumor cells) and positive (proportion of positive tumor cells > 10% of the total tumor cells) subgroups (Figure 1). The evaluation of tumor positivity for a given marker is frequently performed using predetermined standard cutoffs, such as 10% [30,31,32,33,34,35]. The adoption of a categorical scoring system for interpretation simplifies the division of positive and negative groups by pathologists and is further supported by practical observer reproducibility. However, it is assumed that no additional relevant data from the detailed analysis of protein expression of 10–100% will be provided in the production of the results.

### 2.3. Statistical Analyses

Pearson’s chi-square test or Fisher’s exact test were used to evaluate any potential association between SSBP2 expression and the clinicopathological parameters, including age, sex, tumor location, tumor size, histologic grade, lymphatic invasion, vascular invasion, perineural invasion, tumor deposit, tumor budding, and AJCC (American Joint Committee on Cancer) staging. The differences in SSBP2 expression between groups were compared using the Mann–Whitney U test and Wilcoxon signed-rank test. The overall survival (OS) was defined as the duration between the date of curative resection and the date of death or the last follow-up. Recurrence-free survival (RFS) was defined as the duration between curative resection and the date of the first recurrence. Kaplan–Meier survival curves, the log-rank test, and the Cox proportional hazard regression model were used for survival analysis. Two-sided *p*-values < 0.05 were considered statistically significant. All statistical analyses were performed using SPSS version 24.0 (SPSS Inc., Chicago, IL, USA).

## 3. Results

### 3.1. Patient Characteristics and SSBP2 Expression

The median age of patients with colorectal adenocarcinoma and adenoma was 64 years (range, 27–89 years) and 60 years (range, 30–82 years), respectively, and the male-to-female ratios were 1.56:1 and 1:1, respectively. The median follow-up period for the patients in this study was 108 months (range, 1–166 months). Among the 391 colorectal adenocarcinoma patients, 25 (6.4%) patients exhibited metastatic disease at the time of initial diagnosis, 81 (20.7%) patients had recurrence at the time of analysis, and 154 (39.4%) patients had died at the time of analysis.

**Figure 2:**
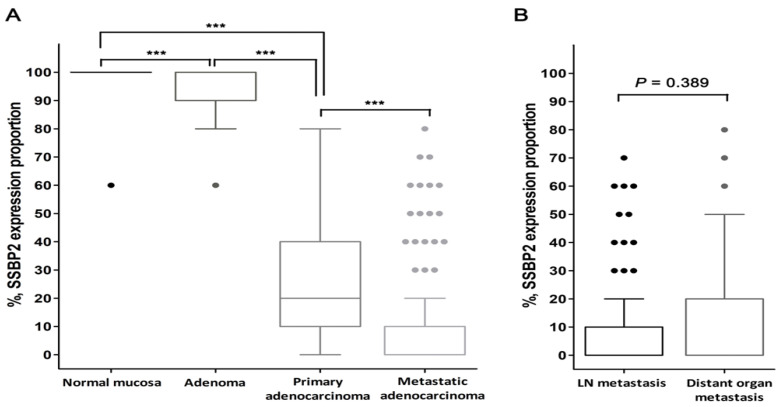
Box and whisker plots of SSBP2 expression proportion. (**A**) SSBP2 expression was significantly decreased with cancer progression (*** *p* < 0.001). (**B**) There was no significant difference between the lymph node metastasis and distant organ metastasis groups. (Horizontal line in the middle of each box, median; boxes, 25 percentile~75 percentile; whiskers, 1.5 × interquartile range from each boundary of the boxes; circles, outlier values with corresponding case number; *p*-value using Mann–Whitney U test, two-tailed.)

Overall, a loss of nuclear SSBP2 expression was observed in 134 (34.3%) primary colorectal adenocarcinoma and 100 (76.3%) metastatic carcinoma tissues, while all normal colonic mucosa and adenoma tissues showed a positive SSBP2 expression. The mean values (%) of SSBP2 expression were 99.17, 94.47, 25.38, and 9.92, respectively. SSBP2 expression was significantly decreased in primary adenocarcinoma and metastatic carcinoma tissues (all, *p* < 0.001; Table 1, Figure 2A). There was no significant difference between the lymph node metastasis and distant organ metastasis groups (*p* = 0.389; Figure 2B). Among the 39 distant organ metastasis cases, 30 affected the liver, six affected the lungs, and three affected the ovaries; SSBP2 expression was significantly lower in the liver than in other organs (mean value = 7.3% vs. 32.2%, *p* = 0.011). A paired-samples Wilcoxon signed-rank test was conducted to compare SSBP2 expression in distant organ metastatic tissues and matched primary CRC tissues. There was a significant difference in the expression proportion (mean value = 32.7% vs. 16.8%, *p* = 0.008).

### 3.2. Correlation between SSBP2 Expression and Clinicopathological Features

To assess the correlation between SSBP2 expression and the clinicopathological parameters, SSBP2 expression was evaluated in 391 primary colorectal adenocarcinomas. The loss of SSBP2 expression was more frequently observed in tumors with vascular invasion (*p* = 0.005) and a high pT category (*p* = 0.045). There was no significant correlation between SSBP2 expression and other clinicopathological parameters (Table 2).

### 3.3. Prognostic Significance of SSBP2 Expression

**Figure 3:**
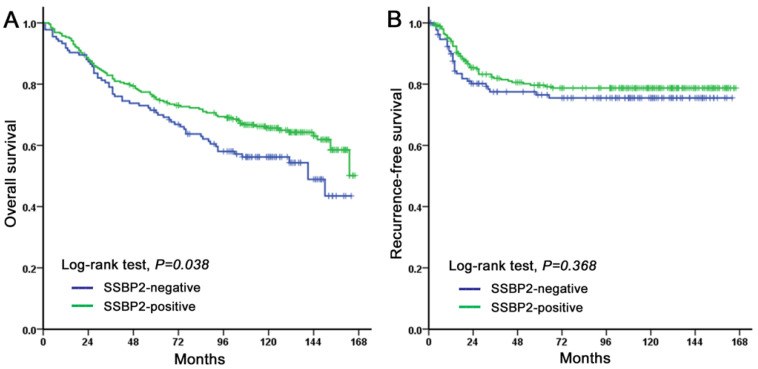
Kaplan–Meier analysis of SSBP2 expression in CRC patients. Overall survival (**A**) was shorter in patients showing a loss of SSBP2 expression compared to those showing a positive SSBP2 expression. Recurrence-free survival (**B**) was not associated with SSBP2 expression.

The OS of CRC patients with a loss of SSBP2 expression was significantly shorter (*p* = 0.038, Figure 3A). However, SSBP2 expression did not affect the RFS (*p* = 0.368, Figure 3B). The univariate survival analysis for OS showed that SSBP2 expression, age, sex, pT category, nodal status, stage, histologic grade, lymphatic invasion, vascular invasion, perineural invasion, and tumor budding were significantly associated with OS (*p* < 0.05 for all cases) (Table 3). Multivariate Cox regression analysis, including SSBP2 expression, age, sex, pT category, nodal status, histologic grade, lymphatic invasion, vascular invasion, perineural invasion, and tumor budding, revealed that age (*p* < 0.001), sex (*p* = 0.004), and vascular invasion (*p* < 0.001) were independent prognostic factors for a poor OS, while SSBP2 expression was not statistically significant (Table 3).

## 4. Discussion

In this study, we showed that SSBP2 expression is significantly associated with OS and poor prognostic factors, such as vascular invasion and a high pT category.

SSBP2 is downregulated in several malignancies, such as esophageal squamous cell carcinoma, prostate cancer, and acute myeloid leukemia, and in previous studies, it has been speculated that promoter methylation may be the main mechanism of loss of SSBP2 expression. Recent studies have revealed that SSBP2 is one of the genes that are downregulated by the methylation pathway [18,20,21,24,36]. In 2011, Huang et al. compared SSBP2 methylation in normal and tumor tissues in 20 pairs of esophageal squamous cell carcinoma and matched normal esophageal tissues using TaqMan-MSP analysis, and a higher degree of SSBP2 methylation in paired tumors than in paired normal tissues was observed in 15 of 20 esophageal squamous cell carcinoma patients [18]. Jun-Wei et al. reported that the SSBP2 promoter region was hypermethylated in 61.4% (54 of 88) of prostate cancer cases, whereas none of the 23 benign prostatic hyperplasia cases showed hypermethylation [21]. They further examined the SSBP2 expression pattern using immunohistochemistry and showed that SSBP2 was significantly downregulated in most of the primary prostate cancer tissues, compared with normal prostate tissues. In addition, another study was conducted to identify a panel of epigenetic biomarkers that can distinguish cholecystitis from gallbladder cancer patients. This study revealed that promoter methylation statuses of SSBP2 (*p* = 0.01) were significantly different in patients with gallbladder cancer when compared to those of patients with cholecystitis [20]. Furthermore, in ovarian cancer, SSBP2 methylation was found in 9% of tumor cases, whereas no cases showed methylation of the SSBP2 promoter in normal tissues [37].

The prognostic impact of SSBP2 expression in CRC has been mentioned in only a few studies. Shannon et al. reported that patients with a lower expression of SSBP2 were potentially correlated with a poorer OS and a shorter DFS, compared to those with a higher expression, using IHC. However, these results were not statistically significant. According to the article, the authors determined the staining results semi-quantitatively based on the staining intensity and percentage of stained tumor cells. Although patients with a lower expression of SSBP2 potentially have a poorer OS and DFS, they did not reach statistical significance (median OS, 29.8 months vs. 42.3 months for a low and high expression, respectively, HR 1.886, 95% CI 0.812–4.378, *p* = 0.140; median DFS, 14 months vs. 30 months for a low and high expression, respectively; HR 1.913; 95% CI, 0.825–4.437; *p* = 0.131) [27]. Perilli et al. studied the changes in expression of transcription factors, such as *HIST1H2BH*, *NABP1* (also known as *SSBP2*), *RND3*, and *TRIO* genes after miR-182 silencing of oncogenic miR-182. As a result, all of them showed a significant upregulation, as confirmed using transcript-specific qRT-PCR assays. In particular, the *SSBP2* gene showed a remarkably high expression in the anti-miR-182-treated tumorigenic cell line. Interestingly, they also reported that a significant decrease in the SSBP2 mRNA levels was identified in primary CRC samples compared to matched normal colon mucosa samples [28].

In this study, we found that the SSBP2 expression pattern is correlated with the OS and several poor clinicopathological factors of the patient. An SSBP2 expression loss was found in 34.3% of primary adenocarcinoma and 76.3% of metastatic adenocarcinoma tissues; however, no expression loss was found in matched normal colonic mucosa and adenoma cases. These results suggested that the loss of SSBP2 expression is possibly associated with aggressive clinical behavior of CRC.

In addition, it is meaningful to combine the results of the previous study [28]. Although Perilli et al. conducted a study with many other transcription factors besides SSBP2, only in the case of SSBP2 it was reported that the mRNA level was markedly reduced in colorectal cancer compared to normal tissues. This result is consistent with the present study, which performed immunohistochemical staining using SSBP2 antibody. This can be positively evaluated in that it suggests the possibility of performing a prognosis-related test for colorectal cancer by a simple method such as immunohistochemistry.

Further studies on SSBP2 promoter methylation and its association with SSBP2 expression in a large cohort of CRC patients using fresh tissues are necessary to understand the mechanism underlying SSBP2-related carcinogenesis. The findings of our study lay the foundation for designing future studies. If the same results are gathered in more groups in the future and target therapy studies for SSBP2 are progressed, it will be a cornerstone for the opening of a new treatment for colorectal cancer, which remains a challenge.

## 5. Conclusions

In conclusion, we showed that SSBP2 expression was significantly decreased in colorectal adenocarcinoma and metastatic carcinoma tissues and was associated with poor prognostic factors. SSBP2 acts as a tumor suppressor and may be used as a prognostic biomarker in colorectal cancer.

## Figures and Tables

**Table 1:** Expression pattern of SSBP2 in normal colonic mucosa, adenoma, primary adenocarcinoma, and metastatic adenocarcinoma.

SSBP2 Nuclear Expression	Normal Mucosa (*n* = 48)	Adenoma (*n* = 47)	Primary Adenocarcinoma (*n* = 391)	Metastatic Carcinoma (*n* = 131)	*p*-Value
Positive	48 (100.0 %)	47 (100.0 %)	257 (65.7 %)	31 (23.7%)	<0.001 ^a^
Negative	0 (0.0%)	0 (0.0 %)	134 (34.3 %)	100 (76.3%)	
Positive cell proportion, % (Mean ± SD)	99.17 ± 5.77	94.47 ± 7.96	25.38 ± 20.80	9.92 ± 18.46	<0.001 ^b^

^a^ Two–sided Pearson’s chi–square test; ^b^ Mann–Whitney U test. Abbreviations: SD, standard deviation.

**Table 2:** SSBP2 expression and clinicopathological parameters in colorectal adenocarcinoma patients (*n* = 391).

Parameters	SSBP2-Negative (*n* = 134)	SSBP2-Positive (*n* = 257)	*p*-Value
Age			0.507
<65 years	72 (35.8%)	129 (64.2%)	
≥65 years	62 (32.6%)	128 (67.4%)	
Sex			0.924
Male	82 (34.5%)	156 (65.5%)	
Female	52 (34.0%)	101 (66.0%)	
Tumor location			0.062
Right side	19 (24.1%)	60 (75.9%)	
Transverse and Left side	13 (30.2%)	30 (69.8%)	
Rectosigmoid	102 (37.9%)	167 (62.1%)	
Histologic grade			0.061
G1 (well differentiated)	7 (24.1%)	22 (75.9%)	
G2 (moderately differentiated)	55 (30.1%)	128 (69.9%)	
G3 (poorly differentiated)	72 (40.2%)	107 (59.8%)	
Lymphatic invasion			0.116
Absent	54 (30.2%)	125 (69.8%)	
Present	80 (37.7%)	132 (62.3%)	
Vascular invasion			0.005
Absent	98 (31.0%)	218 (69.0%)	
Present	36 (48.0%)	39 (52.0%)	
Perineural invasion			0.123
Absent	62 (30.7%)	140 (69.3%)	
Present	72 (38.1%)	117 (61.9%)	
Tumor budding			0.795
Absent/Low/intermediate-grade (0∼9 buds/×200)	79 (33.3%)	155 (66.2%)	
High-grade (≥10 buds/×200)	55 (35.0%)	102 (65.0%)	
T category			0.045
pT1 and pT2	18 (24.3%)	56 (75.7%)	
pT3 and pT4	116 (36.6%)	201 (63.4%)	
Nodal status			0.119
Negative	51 (30.0%)	119 (70.0%)	
Positive	83 (37.6%)	138 (62.4%)	
Stage *			0.119
I	13 (22.8%)	44 (77.2%)	
II	37 (33.3%)	74 (66.7%)	
III	72 (36.4%)	126 (63.6%)	
IV	12 (48.0%)	13 (52.0%)	

* AJCC 8th edition.

**Table 3:** Univariate and multivariate Cox regression analysis for overall survival in colorectal adenocarcinoma patients (*n* = 391).

	Univariate Analysis			Multivariate Analysis		
Variables	HR	95% CI	*p*-Value	HR	95% CI	*p*-Value
SSBP2 (positive vs. negative)	1.406	1.017–1.944	0.038	1.190	0.850–1.666	0.311
Age (<65 vs. ≥65)	2.810	1.996–3.956	<0.001	3.196	2.261–4.518	<0.001
Sex (Female vs. Male)	1.508	1.076–2.114	0.017	1.643	1.167–2.312	0.004
pT category (1,2 vs. 3,4)	1.036	1.013-1.060	0.002	1.015	0.991-1.041	0.225
Nodal status (negative vs. positive)	1.046	1.017-1.075	0.002	0.975	0.889-1.070	0.597
Stage * (I, II vs. III, IV)	1.025	1.010-1.041	0.001			
Histological grade (1,2 vs. 3)	1.664	1.210-2.289	0.002	1.384	0.975–1.964	0.069
Lymphatic invasion (absent vs. present)	1.769	1.273–2.456	0.001	1.571	0.520–4.749	0.424
Vascular invasion (absent vs. present)	2.768	1.964–3.901	<0.001	2.220	1.520–3.244	<0.001
Perineural invasion (absent vs. present)	1.805	1.308–2.491	<0.001	1.244	0.845–1.831	0.268
Tumor budding (Absent/Low/intermediate vs. High)	1.452	1.058-1.993	0.021	1.102	0.777–1.562	0.586

* AJCC 8th edition. Abbreviation: HR, hazard ratio; 95% CI, 95% confidence interval.

## Data Availability

All data generated or analyzed during this study are included in this published article.
